# The need for post-publication peer review in plant science publishing

**DOI:** 10.3389/fpls.2013.00485

**Published:** 2013-12-04

**Authors:** Jaime A. Teixeira da Silva

**Affiliations:** P. O. Box 7, Miki-cho post office, Ikenobe 3011-2Kagawa-ken, 761-0799, Japan

**Keywords:** accountability, bias, ethics, editorial responsibilities, integrity, predatory publishing, snub publishing, transparency

The discussion among scientists about the quality of a published paper should be a constant, dynamic process, even beyond the act of publication. A published paper should not be the final step in the half-life of a scientific manuscript and critical analysis, post-publication, through post-publication peer review (PPPR), should be part of a new and dynamic process that should be embraced by scientists, editors, and publishers alike (Hunter, 2012) as one form of ensuring scientific and academic integrity (Teixeira da Silva, 2013a). Traditional scientific publishing relies primarily on a three-step process: (1) submit; (2) peer review and edit; (3) publish. However, each of these steps has clear documented problems. The first problem related to step 1 involves the intrinsic honesty of the scientist and is the basis upon which the success of all ensuing publishing steps depend. Issues such as appropriate authorship, correct data representation and its faithful representation without manipulation all form part of the first requirement. The fact that this base of honesty has been breached in many instances has forced publishers to insist on increasingly complex signed declarations upon submission of a paper pertaining to the originality of data, the single nature of submission, and conflicts of interest (COIs). Up until submission, trust and honesty lie in the hands of scientists and authors. Apart from such signed declarations, it is rare for publishers to run detailed background checks on authorship, affiliations, or COIs prior to peer review primarily because such aspects are difficult and time-consuming to investigate or verify, especially with a global authorship. More recently, publishers tend to run detailed checks on plagiarism or duplication as a result of more data-bases and stronger web-search engines, but this may fail to reveal duplicate submissions. Therefore, although there has been an increase in the level of verification by publishers in the first step, it is still far from being a fail-safe system. The moment a publisher receives a manuscript for peer review, responsibility is transferred from the scientist to the editors and the publisher (Teixeira da Silva, 2013b). Unlike step one, in which trust was earned (from the author) by the publisher, in step 2, trust is now earned (from the publisher) by the author and the scientific community. Assuming that the author has been honest in step 1, the author would expect some basic responsibilities by the publisher, but practically speaking most likely by the editor-in-chief (EIC) and/or editor board and peer reviewers. Such responsibilities would primarily include: (a) an unbiased peer review (Chase, 2013) of the paper within a reasonable amount of time which should ideally involve a double-blind review in which the identity of the authors is unknown to peer reviewers and *vice versa* to avoid potential COIs; (b) the ability to protect personal information during the peer review; (c) the ability to implement quality control (QC) related to various issues (data, language, structure, literature representation) and to ensure that all peer and editorial requirements made of authors are met.

Regarding the third and last aspect, misrepresentation of the literature, or lack of a strict control of the published literature on the part of authors and editors, has led to the establishment of a new concept, snub publishing (Teixeira da Silva, 2013b). A first-ever case study in the plant sciences involving a PPPR of the *Anthurium* tissue culture literature deserves particular attention since it reveals how the loss of honesty and/or QC can result in the “academic corruption” of the literature, thus weakening the trust in its findings (Teixeira da Silva, 2013c, in press). Finally, once the peer review has been completed, and the paper has been accepted for publication, the responsibilities of the editors, EIC, or publisher do not end there. Accurate representation of the final data set, orderly and structured display of tables and figures lie exclusively in the hands of the publisher, even when authors have been sent a proof. Debate regarding the costs of publishing, intellectual property, and open access (OA) vs. traditional publishing, although important, is marginal to the responsibilities focused on in this paper. However, central to the success of PPPR would be the unfettered access of the public and scientists to a published work for critical analysis.

Without in fact considering issues such as metrics or the debate of the impact factor, which add “noise” to the *de facto* quality of a scientific paper, only two key aspects count when discussing the quality of a paper: (a) the originality of the data set and study; (b) the ability of the author–editor–peer triad to detect and correct as many errors as possible to ensure academic integrity. Understandably, different levels of research and of QC by peer reviewers, editors, or publishers from different cultures may lead to multiple interpretations of issues related to publishing quality and/or ethics such as authorship, self-plagiarism, or duplication. However, reliability of and responsibility associated with the traditional three-step publishing process can be eroded or lost. How then does the scientific community correct or improve the scholarly record once the publishing process has traditionally been perceived to be complete, i.e., a published paper? Once again, this relies on the responsibility of the authors, editors, peers, and publishers. If this first step of the process is flawed, then most likely all ensuing latter steps of QC will also be flawed. For example, a scientist that has falsified data might not necessarily be honest or forthright about their dishonesty, even if errors are discovered in a PPPR. Or an editor, EIC or peer reviewer that has shown bias or poor QC during the peer review process might not wish to admit—following PPPR—to error and failure in the process of academic QC. Finally, a publisher that has assumed that all aspects leading up to the publication of a paper have been well conducted, namely ethical and academic standards, either because it was led to believe that integrity was in place, or because it wanted to believe that such integrity existed, might not be willing to assume responsibility for the entire process for which in fact it was originally responsible. By doing so, a publisher would in essence have to allow an across-the-board PPPR of any paper published in its journal fleet which could overload an already strained publishing process. How then can errors, fraud, or bias be judged if the key players are part of the problem?

The only realistic solution is through PPPR. Although still at a nascent phase for plant science, PPPR can and should only be conducted by specialists in the field. This would allow for a peer who should be independent of the entire process related to a paper's publication history and is thus not be influenced by bias, to critically judge some or all aspects of that paper that have undermined QC as relates to the traditional publishing process. What the PPPR reviewer in fact does is to step in to cover the QC gaps that the authors, peer reviewers, editors, EICs, or publishers have in fact failed to address. Understandably, a paper from a predatory OA publisher might present multiple linguistic, academic, or scientific errors (Bohannon, 2013) relative to a paper published in leading, respected, and/or established plant science journals. Yet, the need to correct the literature must always exist. Depending on the number and level of errors and on the proof of partial or full duplication of text (plagiarism), or self-plagiarism of data, text, figures, or tables, all of which weaken the academic integrity of the scientific literature, the publisher has the responsibility to retract a paper, issue an expression of concern or an *erratum*, even if the authors are in disagreement. The true risk to academic and publishing integrity ultimately lies when non-academic or scientifically unsound literature is referenced by scientists in other scientific papers. When the level of errors is limited, or where issues, concepts, or data are open to debate or multiple interpretation, a PPPR challenge should also be published alongside the original paper (e.g., an OA PDF file) or as a letter to the editor. Ideally, such a PPPR should include the PPPR criticisms or queries, including verifiable proof, a response to those claims by the authors, and the formal position of the EIC and/or editors and publisher. To be fair and balanced, where data is in question, original data sets should be published as annexes, as should the original peer reviewers' reports.

Even though there are still extremely few retractions in the plant science literature, there are still no rules or precedents for PPPR in plant science publishing. This concept should, however, be rapidly accepted by the plant science community and integrated as an established industry norm. This will involve a concerted effort by peers to dedicate freely of their time to carefully scrutinize the *minutiae* of published papers that would be based on a conscientious desire to correct the scientific literature. Ideally, PPPR reports should involve line-by-line or even section-by-section analyses, wherever warranted, rather than a report with broad comments and/or unspecific statements that do not provide detailed enlightenment about the actual errors or weaknesses. All parties should be given a fair opportunity and sufficient time to assess the claims and to respond to them. In cases where the publisher is reticent, the authors are uncooperative or the independent peer does not feel safe throughout the PPPR process, other alternatives exist. One, the subject of both praise and criticism, is anonymity. Using an anonymous report that is supported exclusively by facts, an anonymous PPPR reviewer can expose problems of a paper to authors, the EIC, editors, and publishers without the fear of reprisals (physical, professional, or psychological) by any of the parties the reviewer is questioning. It is then incumbent on the latter three parties to take such reports seriously and to launch and complete a detailed investigation into the claims. Incorrect challenges or misinterpretation by the PPPR should also be noted. Where the parties involved are unresponsive, alternative forms of PPPR are possible, preferably in OA format: (a) independent, self-published critiques; (b) scientific forums such as Retraction Watch or other similar blogs, PubPeer, or the new prototype PubMed Commons. Such forums for open critique of the literature will no doubt increase over time as PPPR becomes the new norm.

The trend is absolutely clear. Unless peers who feel strongly about errors in the scientific literature step forward and make a pro-active, concerted effort, unless publishers who claim ethical standards and peer review support the notion of open-ended publishing and QC through PPPR, and unless we embrace, as plant scientists, that there are serious problems in plant science and in plant science publishing, and that academic and ethical errors in the literature need to be urgently addressed, the efforts of those to publish good, honest, and valuable research will very rapidly be dwarfed by the ever-expanding pool of fraud and/or false information that is becoming increasingly abundant in the literature, but as yet poorly critiqued and quantified.
